# Association analysis between the tag single nucleotide polymorphisms of *DENND1A* and the risk of polycystic ovary syndrome in Chinese Han women

**DOI:** 10.1186/s12881-019-0945-1

**Published:** 2020-01-15

**Authors:** Ya-nan Zhu, Yi-ting Zhang, Qin Liu, Shan-mei Shen, Xiang Zou, Yun-xia Cao, Wen-jun Wang, Long Yi, Qian Gao, Wei-dong Yang, Yong Wang

**Affiliations:** 1 Department of Endodontics, Nanjing Stomatological Hospital, Medical School of Nanjing University, Nanjing, Jiangsu China; 2 State Key Laboratory of Analytical Chemistry for Life Science & Jiangsu Key Laboratory of Molecular Medicine, Medical School, Nanjing University, Nanjing, Jiangsu China; 3 Divisions of Endocrinology, Drum Tower Hospital, Medical School of Nanjing University, Nanjing, Jiangsu China; 4 Department of Health Technology and Informatics, Faculty of Health and Social Sciences, The Hong Kong Polytechnic University, Hong Kong, China; 5 Department of Obstetrics and Gynecology, Anhui Medical University, Hefei, 230022 People’s Republic of China; 6 Centre of Reproduction, Department of Obstetrics and Gynecology, Memorial Hospital of Sun Yat-Sen University, Guangzhou, 510120 People’s Republic of China

**Keywords:** Polycystic ovary syndrome, Genetic polymorphism, *DENND1A*

## Abstract

**Background:**

The *DENND1A* gene is one of the most important sites associated with polycystic ovary syndrome (PCOS). We attempted to analyze the correlation between five single nucleotide polymorphisms (SNPs) in the *DENND1A* gene and the development of PCOS.

**Methods:**

A total of 346 PCOS patients and 225 normal ovulatory women were involved in the case-control study. Clinical variables and hormones were recorded. According to the Hap Map database, five tagging SNPs (rs2479106, rs2768819, rs2670139, rs2536951 and rs2479102) in the *DENND1A* gene were identified. The TaqMan probe and the PCR–RFLP (restriction fragment length polymorphism) methods were used for revealing these genotypes. TaqMan Genotype Software was used to analyze the alleles of the five SNPs.

**Results:**

Linkage disequilibrium and the gene frequency analysis demonstrated that the CCGGG haplotype might increase the risk of PCOS (*P* = 0.038, OR = 1.89, 95% CI = 1.027–3.481). Significant differences were found in genotypic and allelic distributions at the rs2536951 and rs2479102 loci between PCOS women and controls (*P* <  0.001). The LH levels and LH/FSH ratios were higher in PCOS patients than in the control group. A detailed analysis revealed that for the rs2479106 locus, these two values were significantly different in the control subjects who had AA, AG and GG genotypes (*P* = 0.013 and *P* = 0.007, respectively), and for the rs2468819 locus, these two values were significantly different among the PCOS patients with AA, AG and GG genotypes (*P* = 0.013 and 0.002, respectively).

**Conclusions:**

The tagging SNPs rs2479106 and rs2468819 in the *DENND1A* gene are associated with PCOS in the Chinese population, whereas rs2670139, rs2536951 and rs2479102 are not correlated with PCOS in the same population.

## Background

Polycystic ovary syndrome (PCOS) is a highly complex gynecological endocrine disease affecting up to 10% of women of reproductive age [1]. It is the most common endocrine disorder in the gynecological and endocrinological clinic in China, where 50% of patients suffer from PCOS [2, 3]. The clinical manifestations of PCOS can affect many organs, including the hypothalamus, pituitary, ovary, adrenal gland, and pancreas, leading to a higher degree of genetic heterogeneity. The loose ovulation and endocrine disorder are the main reasons for female infertility, which severely compromises women’s physical and mental health. Furthermore, PCOS may be accompanied by an increased risk of diabetes mellitus, glucose intolerance, hypertension, atherogenic dyslipidemia, non-alcoholic fatty liver disease, systemic inflammation and coagulation disorders. The etiology of PCOS has not been clearly defined. It is believed that genetic factors may play important roles in its pathogenesis, and at least 70 candidate responsible genes have been identified [4]. A milestone event for the first attempt of a genome-wide association study (GWAS) on PCOS focused on chromosomes 2p16.3, 2p21 and 9q33.3 in Han Chinese women. The corresponding gene loci were *LHCGR* (luteinizing hormone/choriogonadotropin receptor), *THADA* (thyroid associated protein) and *DENNDIA* (DENN/MADD domain-containing 1A) [5].

Two years later, another GWAS project suggested eight new candidate risk loci for the development of PCOS in the Chinese population. These loci, including follicle stimulating hormone receptor (*FSHR*), *C9orf3, YAP1, RAB5B, HMGA2, TOX3,* insulin receptor (*INSR*), and *SUMO1P1* [6], are found to be associated with PCOS etiology by being involved in the synthesis of reproductive hormones, functional regulation of gonadotropin and insulin resistance [7]. Another large GWAS was recently published focusing on European subjects [8]. The strongest associations in Europeans are found to be in *DENND1A* and *THADA* loci, and additional associations have also been revealed at loci containing *LHCGR, RAB5/SUOX, FSHR* and *YAP1* [9]. These studies prompted us to search for more loci that might account for the origin and pathophysiology of PCOS. Various genetic polymorphisms have been described for PCOS [10], and the association of single nucleotide polymorphisms (SNPs) with the occurrence and development of PCOS has been confirmed. Three SNPs (rs13405728 in *LHCGR* gene; rs13429458 in *THADA* gene and rs2479106 in *DENND1A* gene) have been identified to be genetic variants of PCOS by GWAS in Han Chinese populations [11, 12] .

Among those SNP loci that may have an impact on PCOS, the *DENND1A* gene, one of the *DENND* family members, has attracted substantial attention. *DENND1A* is one of the causal factors expressed in the theca follicle [13]. *DENND1A* SNPs are associated with metabolic disturbances and endocrine disorders [14]. They also have a profound impact on the establishment of hyperandrogenic PCOS phenotypes [15]. *DENND1A*, which encodes the protein connecdenn 1, consists of 22 exons and extends over 500,000 bases. *DENND1A* does not facilitate endocytosis and receptor-mediated turnover. Connecdenn 1, one of the proteins encoded by the *DENND1A* gene, facilitates these functions in the lipid bilayer. It has a clathrin-binding domain that is localized in the N-terminus of the protein and is associated with the metabolism of phosphoinsitol-3-phosphates and other lipids [16]. *DENND1A* is important in facilitating endocytosis and receptor-mediated turnover [17, 18]. DENN domain proteins form a new class of membrane transporters which regulate Rab GTPases [16]. On the other hand, *DENND1A*-encoded domain binds *ERAP1* (endoplasmic reticulum amino acid peptidase 1) as a negative regulator. *ERAP1* expression is elevated in the serum of obese PCOS patients [19]. We speculated that *DENND1A* might affect the pathogenesis of PCOS through dysregulation of *ERAP1* in those PCOS patients with a risk allele of *DENND1A*.

The *DENND1A* gene is associated with PCOS in both Han Chinese and European women, although the associations are focused on different SNPs [20–22]. Some SNPs in the *DENND1A* gene, including rs10818854, rs2479106 and rs10986105, are reported as susceptible loci [12, 14]. Among the many polymorphisms of *DENND1A* genes, rs2479106 and rs10818854 polymorphisms have received much attention. Several studies have previously suggested that the rs2479106 and rs10818854 polymorphisms are associated with an increased risk of PCOS [23, 24].

In addition to the rs2479106 and rs10818854 polymorphisms, other SNPs related to the physical signs and disease characteristics of PCOS might also exist. In this study, The LD pattern of the *DENND1A* gene including almost all the SNPs is presented in a full picture based on the Hap Map Phase III Han Phase database in NCBI by Hap lo View software version 4.2. Based on the LD pattern, we attempted to select a few SNPs (with linkage and representation) to determine whether polymorphisms of the *DENND1A* gene were related to PCOS, both at the individual SNP and haplotype levels, in the Han Chinese population. As most studies suggest that the polymorphism site rs2479106 in the *DENND1A* gene is related to PCOS susceptibility, we chose the other four SNPs which have a strong relation with rs2479106. Finally, five tagging SNPs including rs2479106, rs2768819, rs2670139, rs2536951 and rs2479102 were selected as the representative loci for investigating the association between the tag SNPs of *DENND1A* and the risk of PCOS in Chinese Han women.

## Methods

### Subjects

Using the 2003 Rotterdam Criteria (The Rotterdam ESHRE/ASRM-Sponsored PCOS Consensus Workshop Group, 2004), we recruited 346 PCOS patients and 225 women without PCOS, both being the Chinese Han women. The samples were collected from subjects recruited from Drum Tower Hospital in Nanjing, Department of Obstetrics and Gynecology, Anhui Medical University in Hefei, and Memorial Hospital of Sun Yat-Sen University, Guangzhou in China. Patients with PCOS were diagnosed according to the 2003 Rotterdam Criteria (The Rotterdam ESHRE/ASRM-Sponsored PCOS Consensus Workshop Group, 2004). The Rotterdam Criteria require at least two of the following indicators for diagnosis of PCOS: (1) Oligo- and/or anovulation; (2) Clinical and/or biochemical signs of hyperandrogenism; and (3) Polycystic ovaries and exclusion of other aetiologies (congenital adrenal hyperplasias, androgen-secreting tumors, Cushing’s syndrome).

The women in the control group came to visit the clinic for other reasons (such as tubal factor infertility or their husbands’ infertility). Some of them had given birth to one child or more. Their menstrual cycles were normal (< 32 days) and exclusion criteria were hirsutism, insulin resistance, other property of hyperandrogenism and obesity.

The study was approved by the Medical School of Nanjing University. The patients and the control women provided informed consent and volunteered to participate in the study.

### Clinical and biochemical determination

First, clinical variables were recorded, including height and body weight. Body mass index (BMI) was calculated as weight (kg) divided by height (m). Second, we collected the peripheral blood samples between 8 am and 9 am after a 12-h overnight fast. For those women in the menstrual cycle, blood samples were collected from the 3rd day to the 5th day. For those women who had amenorrhea, blood samples were collected at a random day. A series of biochemical measurements were carried out to obtain the hormone levels, including luteinizing hormone (LH), total testosterone (T), follicle-stimulating hormone (FSH) and estradiol (E2) using radioimmunoassay (Beijing North Institute of Biological Technology, China).

### Characterization of linkage disequilibrium (LD) and selection of tag SNPs

**Fig. 1:**
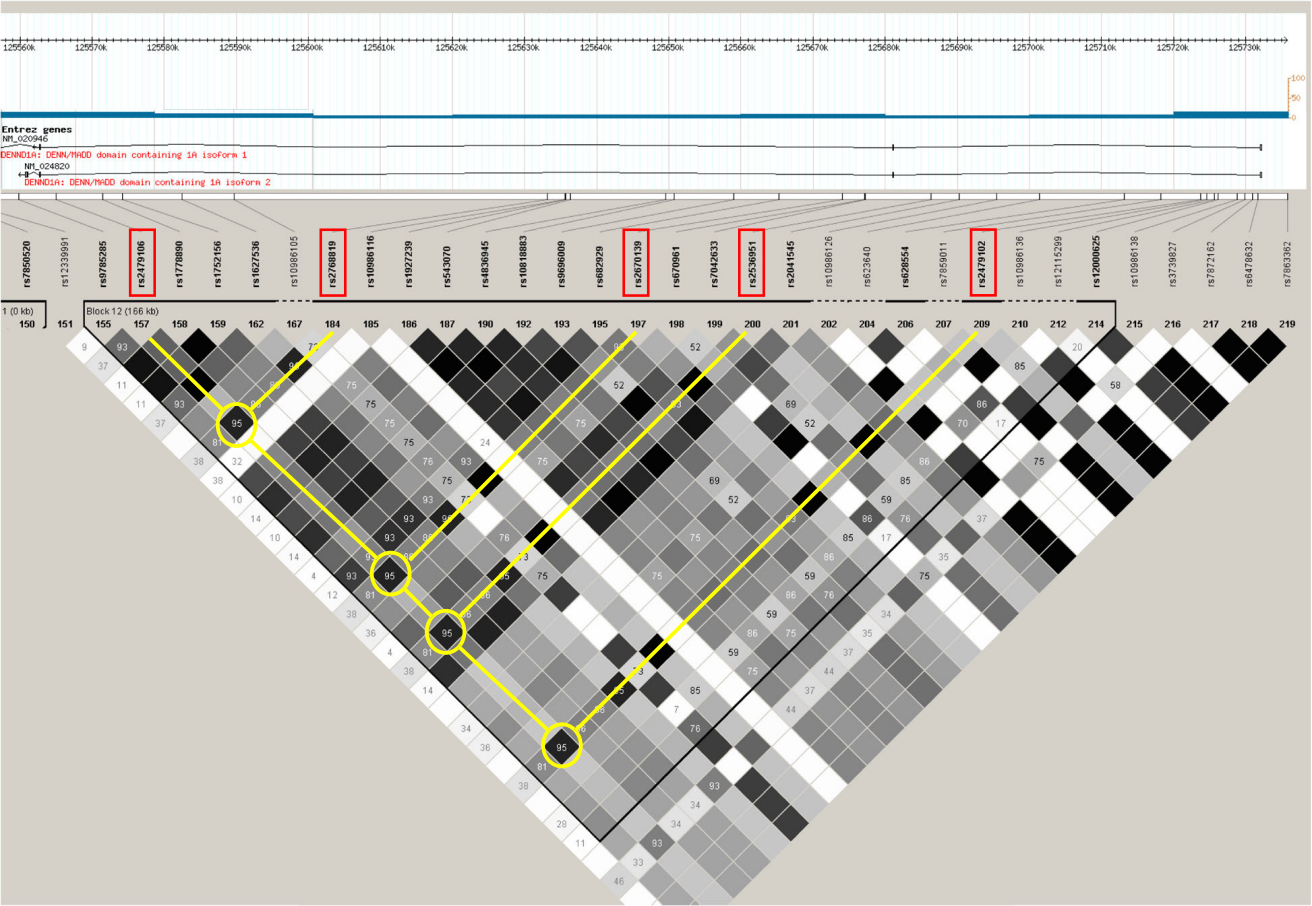
Block 12, a section taken from the full picture of the LD pattern of *DENND1A* gene based on the Hap Map database (www.hapmap.org, Hap Map database release no. R2/phase III, population: CHB) for the Chinese Han population. Five strong linkage SNPs we chose for the following association study (rs2479106, rs2768819, rs2670139, rs2536951 and rs2479102) were marked in red box. The loci of rs2479106 was selected as those references. The other four SNPs were selected for high linkage correlations with rs2479106 (measured as D’ = 95%)

The *DENND1A* gene is located on chromosome 9q33.3. The SNPs genotyped were tagging SNPs in the *DENND1A* gene based on the HapMap database (www.hapmap.org, HapMap database release no. R2/phase III, population: CHB) for the Chinese Han population (Additional file 1: Figure S1–1, S1–2). The site rs2479106 and the other four SNPs were selected with strong linkage in Block 12. The pair-wise correlations between rs2479106 and these SNPs were the same (measured as D’ = 95%). Finally, the five tagging SNPs, rs2479106, rs2768819, rs2670139, rs2536951 and rs2479102, were selected for the following association study. (Fig. 1).

### Genotyping for polymorphisms

Genomic DNA was extracted from blood samples with an SBS UltraPure™Genome DNA kit (SBS Genentech, Shanghai, China) and examined by a Thermo Scientific Nano Drop™ 2000 spectrophotometer (Thermo Electron, Massachusetts, USA). Genomic DNA was stored at 20 ng/μl in ultrapure water and at − 80 °C until further genotyping analysis for polymorphisms by the TaqMan SNP genotyping assay. PCR amplification was conducted in a total volume of 10 μl composed of 5 μl 2X TaqMan Master Mix, 0.5 μl 20X Assay Working Stock, 1 μl sample Genomic DNA (20 ng per well), and 3.5 μl nuclease-free water by the One Step One Plus RT-PCR System (Applied Biosystems). PCR was carried out as follows: 95 °C Ampli Taq Gold UP Enzyme Activation for 10 min, followed by 40 cycles consisting of 95 °C denaturation for 15 s and 60 °C annealing/extension for 1 min, ending with a single extension of 15 min at 60 °C.

### Statistical analysis

SPSS 17.0 was used for statistical analyses. *P* <  0.05 was considered statistically significant. The age, AAM, BMI and serum hormone level data were presented as the means ± SD. The differences in hormone levels of different genotypes in the two groups were detected by one-way analysis of variance (ANOVA). The genotype data were calculated with the expectation-maximization (EM) algorithm, presented in Haplo View version 4.2, and analyzed by the SHEsis online software (http://analysis.bio-x.cn/myAnalysis.php). Next, it was measured with the pairwise LD analysis. The alleles of the five SNPs were analyzed using TaqMan Genotype Software. The χ^2^ test of the 2 × 3 tables was used to compare the genotypic distributions and Hardy-Weinberg distribution of genotypes between patients and controls. The differences among the three genotypes were presented by the Turkey test.

## Results

### Characterization of the clinical features of PCOS and the controls

Characterization of the clinical and biochemical features of the PCOS patients (*n* = 346) and control women (*n* = 225) are listed in Table 1. PCOS patients were diagnosed under the Rotterdam Criteria. Significant differences in BMI, E2, LH and LH/FSH ratios were revealed between the two groups. Compared with the controls, PCOS patients were characterized by higher BMI, E2, LH and LH/FSH ratios.

**Table 1:** Clinical characteristics of PCOS and control subjects

Groups	N	BMI* (kg/m2)	E2* (pMol/L)	T (nMol/L)	LH* (IU/L)	FSH (IU/L)	LH/FSH*	PRL(IU/L)
PCOS	346	29.69± 3.14	102.19 ± 20.3	2.84± 0.15	9.79± 1.76	6.78± 0.52	1.44 ± 0.03	5.75 ± 1.06
Control	225	22.03± 2.35	60.19 ± 10.91	2.26± 0.32	5.22± 0.91	7.87± 1.06	0.70 ± 0.12	5.96 ± 1.03

*P <  0.05 between the PCOS and control groups. Data are expressed as mean ± standard deviation. *BMI* body mass index, *E2* estradiol, *T* testosterone, *LH* luteinizing hormone, *FSH* follicle-stimulating hormone, *PRL* prolactin

### The LD structure and haplotype-based association analyses

**Fig. 2:**
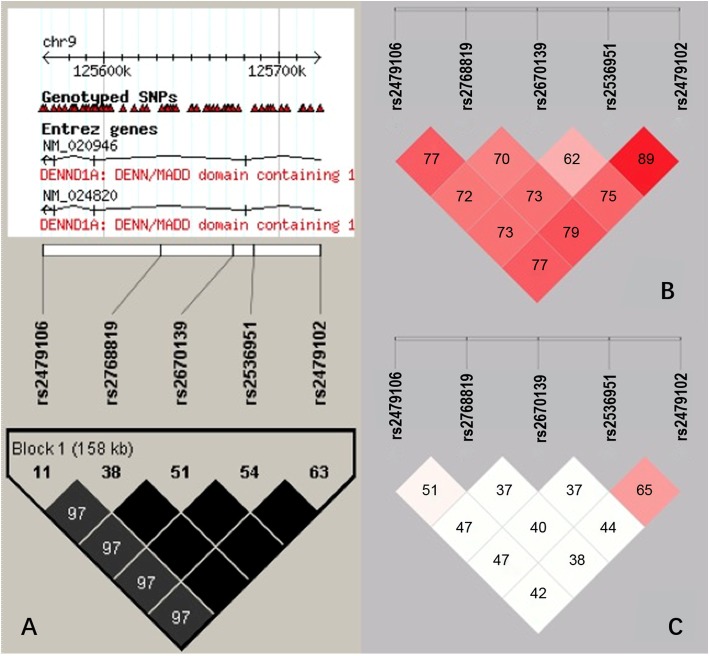
The LD analysis pattern of the five strong linkage SNPs (rs2479106, rs2768819, rs2670139, rs2536951 and rs2479102) selected from the *DENND1A* gene. **a** is the pattern according to database of NCBI for Chinese Han population. It is spanning from 2 kb upstream to 5 kb downstream of *DENND1A* within 125,600 k to125700k. The value within the diamond represents the pair-wise correlation between SNPs (measured as D’). The Hap lo View of LD pattern from the data of our research were presented in (**b**) (measured as D’) and (**c**) (measured as R^2^)

Based on the Hap Map Phase III database (release no. R^2^/phase III, population: CHB), five SNPs were identified in the promoter of the *DENND1A* gene (MAF > 0.05). Five tag SNPs were revealed by Haplo View, through LD analysis and gene frequency analysis according to NCBI (Fig. 2a). The pairwise correlation of two SNPs was indicated as the value in the diamond (measured as D’). The Hap lo View of LD pattern from the data of our research were presented in Fig. 2b (measured as D’) and Fig. 2c (measured as R^2^). On the one hand, these data showed that the 5 SNPs had linkage correlation within each other. On the other hand, these SNPs also have their distinct functions.

LD structures with the genotype data for SNPs were reexamined to obtain haplotype-based association analyses. There was a significant difference of haplotype frequencies in the CCGGG haplotype between the patients and the controls (*P* = 0.0386, OR = 1.891, 95% CI = 1.027–3.481) (Table 2). The CCGGG haplotype might enhance susceptibility to PCOS.

**Table 2:** Correlation between the haplotypes containing the 5 tag SNPs including rs2479106, rs2768819, rs2670139, rs2536951 and rs2479102 and the PCOS risk in cases and controls

	Case (freq)	Control (freq)	Pearson’s *P*	Odds Ratio [95%CI]
CCGGG	19 (0.201)	39 (0.112)	0.038*	1.89 [1.027~3.481]
TTAAA	64 (0.680)	212 (0.602)	0.399	1.26 [0.733~2.176]
TTAGA	3 (0.032)	4 (0.012)	0.207	2.55 [0.567~11.498]

*P <  0.05 between the cases and controls

### Allele distribution and SNP genotype analysis

Distributions of allelic and genotypic frequencies of five SNPs (rs2479106, rs2768819, rs2670139, rs2536951 and rs2479102) are listed in Tables 3 and 4. The distributions of the rs2479106 genotype were 0.3380 for AG, 0.6130 for AA, and 0.0490 for GG. The data of the rs2768819 genotype were 0.3488 for AG, 0.6192 for AA and 0.3198 for GG. The distributions of the rs2670139 genotype were 0.2826 for CT, 0.0917 for CC and 0.6257 for TT. Analyses of rs2536951 resulted in 0.2962 for AG, 0.6044 for AA and 0.0994 for GG. For rs2479102, data analysis pointed to 0.2882 for CT, 0.0961 for CC and 0.6157 for TT. The genotypic distribution did not reveal significant differences for rs2768819, rs2479106 or rs2670139 between PCOS and healthy women (*P* = 0.842, 0.277 and 0.465, for rs2768819, rs2479106 and rs2670139, respectively). No significant differences were found regarding allelic distribution between the two groups (*P* = 0.985, 0.37 and 0.23 for rs2768819, rs2479106 and rs2670139, respectively). Interestingly, significant differences in the genotypic distribution for rs2536951 and rs2479102 between the patients and controls were identified (*P* <  0.001), and similar differences were also found in allelic distribution (*P* <  0.001, Table 3). The distributions of genotypes were in agreement with Hardy-Weinberg equilibrium.

**Table 3:** Genotypes and alleles of rs2479106, rs2768819, rs2670139, rs2536951 and rs2479102 in PCOS cases and controls

rs2479106	Genotypes, n (%)			*p*-value^a^	Alleles, n (%)		*p*-value^b^
	AG	AA	GG		A	G	
Control	78(0.347)	140(0.622)	7(0.031)	0.277	358(0.796)	92(0.204)	0.37
PCOS	115(0.332)	210(0.607)	21(0.061)		535(0.773)	157(0.227)	
rs2768819	Genotypes, n (%)			*p*-value^a^	Alleles, n (%)		*p*-value^b^
	AG	AA	GG		A	G	
Control	31(0.365)	52(0.612)	2(0.024)	0.842	135(0.794)	35(0.206)	0.985
PCOS	89(0.344)	161(0.622)	9(0.035)		411(0.793)	107(0.207)	
rs2670139	Genotypes, n (%)			*p*-value^a^	Alleles, n (%)		*p*-value^b^
	CT	CC	TT		C	T	
Control	65(0.284)	25(0.109)	139(0.607)	0.465	115(0.251)	343(0.749)	0.23
PCOS	89(0.282)	25(0.079)	202(0.639)		139(0.220)	493(0.780)	
rs2536951	Genotypes, n (%)			*p*-value^a^	Alleles, n (%)		*p*-value^b^
	AG	AA	GG		A	G	
Control	67(0.307)	116(0.532)	35(0.161)	< 0.001	299(0.686)	137(0.314)	< 0.001
PCOS	82(0.288)	188(0.660)	15(0.053)		458(0.804)	112(0.196)	
rs2479102	Genotypes, n (%)			*p*-value^a^	Alleles, n (%)		*p*-value^b^
	CT	CC	TT		C	T	
Control	61(0.269)	37(0.163)	129(0.568)	< 0.001	135(0.297)	319(0.703)	< 0.001
PCOS	86(0.304)	12(0.042)	185(0.654)		110(0.194)	456(0.806)	

*P*-value^a^ is based on the genotype frequencies versus control.

*P*-value^b^ is based on the allele frequencies versus control.

**Table 4:** Clinical and metabolic characteristics of genotypes containing rs2479106, rs2768819, rs2670139, rs2536951 and rs2479102 in PCOS cases and controls

Genotypes	Control			*p*-value	PCOS			*p*-value
rs2479106	AG	AA	GG		AG	AA	GG	
Age (years)	33.53 ± 0.65	32.58 ± 0.49	34.40 ± 2.27	0.771	26.97 ± 0.72	26.15 ± 0.60	33.00 ± 0.49	0.227
BMI (kg/m2)	22.72 ± 0.49	21.51 ± 0.26	23.41 ± 0.62	0.143	23.33 ± 0.72	23.06 ± 0.54	19.96 ± 0.49	0.268
E2(pg/ml)	63.06 ± 7.36	57.13 ± 4.40	42.87 ± 5.23	0.436	209.61 ± 25.42	256.54 ± 16.61	102.19 ± 28.39	0.184
T(nm/l)	2.93 ± 0.17	2.04 ± 0.32	1.34 ± 0.32	0.147	3.99 ± 1.06	6.27 ± 2.15	2.84 ± 1.05	0.727
E2/T(ln)	21.33 ± 1.72	72.21 ± 25.10	33.11 ± 2.87	0.335	92.12 ± 12.49	93.92 ± 12.68	35.98 ± 5.49	0.797
LH(IU/l)	5.94 ± 0.64	4.79 ± 0.29	2.56 ± 0.83	0.013*	15.73 ± 1.31	17.11 ± 1.19	9.79 ± 1.48	0.318
FSH(IU/l)	8.40 ± 0.66	7.90 ± 0.31	7.00 ± 0.41	0.701	7.17 ± 0.50	6.27 ± 0.33	6.78 ± 0.29	0.551
LH/FSH	0.78 ± 0.07	0.64 ± 0.04	0.36 ± 0.11	0.007*	2.99 ± 0.19	2.94 ± 0.20	1.44 ± 0.12	0.068
PRL(μg/l)	4.67 ± 1.35	6.31 ± 1.78	1.79 ± 0.03	0.724	14.62 ± 1.59	18.21 ± 2.10	5.75 ± 1.05	0.365
rs2768819	Control			*p*-value	PCOS			*p*-value
	AG	AA	GG		AG	AA	GG	
Age (years)	32.6 ± 0.77	33.58 ± 0.51	34.00 ± 1.00	0.935	28.63 ± 0.84	27.24 ± 0.80	22.5 ± 0.65	0.269
BMI (kg/m2)	22.7 ± 0.59	21.93 ± 0.32	21.69 ± 2.79	0.465	13.52 ± 0.88	23.30 ± 0.79	19.72 ± 0.86	0.678
E2(pg/ml)	62.7 ± 8.65	61.36 ± 5.27	56.36 ± 25.64	0.983	197.51 ± 32.63	192.59 ± 17.67	250.90 ± 30.35	0.899
T(nm/l)	2.71 ± 0.33	2.09 ± 0.14	1.67 ± 0.65	0.339	2.01 ± 0.15	2.55 ± 0.26	3.35 ± 0.64	0.225
E2/T(ln)	20.5 ± 3.37	72.51 ± 10.75	32.75 ± 2.51	0.241	109.08 ± 16.66	75.45 ± 15.55	84.77 ± 17.94	0.917
LH(IU/l)	5.45 ± 0.52	5.19 ± 0.34	4.58 ± 1.41	0.905	11.75 ± 1.26	16.66 ± 1.61	31.70 ± 2.34	0.013*
FSH(IU/l)	6.59 ± 0.28	8.04 ± 0.38	8.42 ± 0.44	0.057	6.58 ± 0.54	7.06 ± 0.50	6.21 ± 0.54	0.779
LH/FSH	0.89 ± 0.09	0.68 ± 0.04	0.54 ± 0.14	0.222	1.85 ± 0.16	2.44 ± 0.21	5.26 ± 0.69	0.002*
PRL(μg/l)	4.58 ± 2.91	7.64 ± 2.18	1.80 ± 0.06	0.583	15.16 ± 1.69	19.53 ± 3.71	24.43 ± 1.33	0.618
rs2536951	Control			*p*-value	PCOS			*p*-value
	AG	AA	GG		AG	AA	GG	
Age (years)	33.3 ± 0.99	32.61 ± 0.52	32.30 ± 0.96	0.667	27.43 ± 0.68	25.98 ± 0.62	30.00 ± 2.00	0.166
BMI (kg/m2)	22.3 ± 0.61	21.71 ± 0.31	21.44 ± 0.80	0.442	23.41 ± 0.79	22.99 ± 0.58	26.89 ± 3.16	0.413
E2(pg/ml)	61.0 ± 6.03	56.40 ± 4.51	59.45 ± 12.17	0.861	217.63 ± 29.31	247.00 ± 19.28	232.68 ± 80.20	0.699
T(nm/l)	2.85 ± 0.37	2.28 ± 0.36	1.09 ± 0.07	0.268	3.43 ± 0.83	4.01 ± 0.48	2.12 ± 0.56	0.67
E2/T(ln)	20.7 ± 9.80	70.70 ± 27.74	28.84 ± 1.41	0.412	93.06 ± 14.93	89.39 ± 12.39	107.24 ± 9.50	0.947
LH(IU/l)	6.19 ± 0.51	5.07 ± 0.38	5.25 ± 0.70	0.284	15.74 ± 1.47	17.21 ± 1.37	12.58 ± 2.02	0.739
FSH(IU/l)	8.10 ± 0.59	8.14 ± 0.37	7.70 ± 0.79	0.93	6.77 ± 0.51	6.36 ± 0.38	7.36 ± 0.71	0.65
LH/FSH	0.88 ± 0.07	0.64 ± 0.04	0.72 ± 0.08	0.026*	2.35 ± 0.17	2.91 ± 0.21	1.75 ± 0.44	0.139
PRL(μg/l)	1.73 ± 0.69	5.09 ± 1.85	3.79 ± 0.63	0.98	13.15 ± 1.55	18.21 ± 2.36	18.33 ± 8.65	0.358
rs2479102	Control			*p*-value	PCOS			*p*-value
	CT	CC	TT		CT	CC	TT	
Age (years)	33.2 ± 0.71	33.40 ± 1.06	32.80 ± 0.50	0.561	27.56 ± 0.74	23.33 ± 2.60	26.60 ± 0.63	0.218
BMI (kg/m2)	22.3 ± 0.55	22.94 ± 1.05	21.64 ± 0.26	0.197	23.40 ± 0.90	20.25 ± 1.88	23.48 ± 0.61	0.443
E2(pg/ml)	62.4 ± 7.30	47.53 ± 4.73	59.70 ± 4.79	0.525	190.30 ± 26.79	215.11 ± 31.41	240.79 ± 20.58	0.35
T(nm/l)	2.75 ± 0.33	1.20 ± 0.10	2.16 ± 0.29	0.191	3.24 ± 0.89	2.48 ± 0.52	3.36 ± 0.45	0.915
E2/T(ln)	21.9 ± 3.88	24.52 ± 3.66	64.40 ± 21.94	0.374	84.89 ± 12.45	89.30 ± 7.27	103.02 ± 14.29	0.702
LH(IU/l)	5.68 ± 0.58	5.06 ± 0.60	5.04 ± 0.35	0.619	14.86 ± 1.61	21.22 ± 5.02	15.56 ± 1.14	0.424
FSH(IU/l)	7.90 ± 0.61	9.02 ± 0.90	7.86 ± 0.34	0.129	6.94 ± 0.47	6.06 ± 0.30	6.32 ± 0.39	0.594
LH/FSH	0.80 ± 0.07	0.59 ± 0.07	0.67 ± 0.04	0.243	2.13 ± 0.18	3.55 ± 0.91	2.72 ± 0.22	0.092
PRL(μg/l)	4.64 ± 3.35	3.79 ± 0.63	5.76 ± 1.64	0.902	13.82 ± 1.57	22.26 ± 7.07	18.52 ± 2.49	0.38
rs2670139	Control			*p*-value	PCOS			*p*-value
	CT	CC	TT		CT	CC	TT	
Age (years)	33.2 ± 0.67	32.79 ± 1.11	32.66 ± 0.51	0.807	27.59 ± 0.71	25.50 ± 2.50	26.12 ± 0.60	0.318
BMI (kg/m2)	22.20 ± 0.5	22.33 ± 1.17	21.61 ± 0.25	0.458	23.40 ± 0.83	21.73 ± 2.01	23.20 ± 0.55	0.857
E2(pg/ml)	57.9 ± 6.98	60.14 ± 12.70	55.72 ± 3.41	0.89	214.98 ± 29.90	201.58 ± 49.11	250.40 ± 17.80	0.536
T(nm/l)	2.81 ± 0.31	2.15 ± 0.75	2.01 ± 0.26	0.198	3.37 ± 0.85	2.46 ± 0.90	6.37 ± 2.04	0.592
E2/T(ln)	22.0 ± 3.89	23.19 ± 7.55	66.16 ± 23.76	0.371	95.37 ± 15.37	86.28 ± 11.45	91.78 ± 11.84	0.979
LH(IU/l)	5.39 ± 1.00	4.84 ± 1.49	5.03 ± 0.35	0.786	14.86 ± 1.51	22.83 ± 8.24	17.63 ± 1.23	0.945
FSH(IU/l)	7.65 ± 1.10	8.23 ± 2.35	8.09 ± 0.36	0.129	6.57 ± 0.47	6.27 ± 0.38	6.73 ± 0.36	0.272
LH/FSH	0.78 ± 0.14	0.63 ± 0.16	0.64 ± 0.04	0.476	2.29 ± 0.19	3.73 ± 1.54	2.83 ± 0.20	0.138
PRL(μg/l)	2.53 ± 0.77	1.79 ± 0.04	4.63 ± 1.42	0.769	12.82 ± 1.37	16.33 ± 6.65	18.17 ± 2.16	0.294

^*^P < 0.05. Statistical analyses were carried out by analysis of covariance to correct for age and BMI. Data are expressed as mean ± standard deviation. *BMI* body mass index, *E2* estradiol, *T* testosterone, *LH* luteinizing hormone, *FSH* follicle-stimulating hormone

### Clinical and metabolic characteristics among the different genotypes

**Fig. 3:**
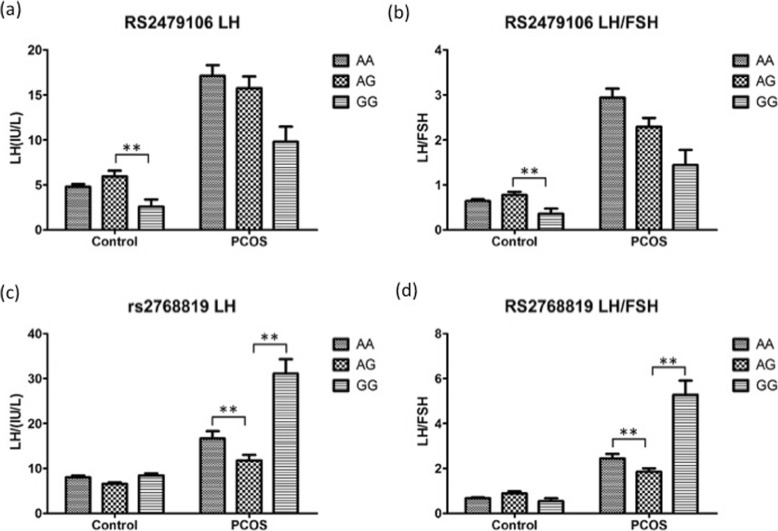
Differential regulation of pathogenic indicators with respect to polymorphisms in rs2479106 and rs2768819. Luteinizing hormone levels (**a**, **c**) and the ratios of luteinizing hormone to follicle-stimulating hormone (**b**, **d**) of PCOS patients and controls were measured and presented with respect to their rs2479106 (**a**, **b**) and rs2768819 (**c**, **d**) genotypes. Data are presented as the means ± SEM. ** *P* < 0.05

Clinical and metabolic characteristics among the different genotypes (AA, AG and GG) of the five SNPs (rs2479106, rs2768819, rs2670139, rs2536951 and rs2479102) in patients and the controls are listed in Table 4. For rs2479106, serum LH levels and the ratios of LH/FSH were significantly different among AA, AG, and GG genotypes in the controls (*P* = 0.013 and *P* = 0.007), while there was no difference in the patients (Fig. 3a, b). LH concentrations and LH/FSH ratios of rs2468819 significantly differed among AA, AG, and GG genotypes in the patients (*P* = 0.013 and 0.002, respectively) (Fig. 3c, d). However, no impact on controls was found. The LH/FSH ratios of rs2536951 demonstrated a significant difference among AA, AG, and GG genotypes in the controls (*P* = 0.026), but there was no difference in PCOS patients. No significant differences were found among AA, AG, and GG genotypes of rs2670139 and rs2479102, in either PCOS patients or controls. There were no significant differences in BMI or levels of any other serum hormones, such as testosterone, prolactin, E2/T, LH, FSH, LH/FSH and estradiol, in the other two genotypes in PCOS patients and controls (Table 4).

## Discussion

In this study, we provided data suggesting that the promoter polymorphism of the *DENND1A* gene could impact the incidence of PCOS, which is consistent with recent reports showing that the rs2479106 G allele is significantly associated with decreased sensitivity to PCOS [25] [26]. A GWAS analysis also resulted in a similar finding [5].

In PCOS women, LH, FSH, LH/FSH ratio and TSH were significantly increased compared with the control group [27]. Subsequent quantitative trait analysis revealed that there was an association between the rs2768819 GG genotype and increased levels of LH as well as a higher LH/FSH ratio in PCOS patents. For the control subjects, higher LH levels and increased LH/FSH ratios were observed in those with the rs2479106 AG genotype. However, additional samples are required to confirm the association at the other haplotype loci.

Evidence of familial aggregation and geographical impact on the clinical traits suggested that the pathogenesis of PCOS involves a variety of genetic and environmental factors. A conditional logistic regression analysis showed that both rs10818854 and rs2479106 are associated PCOS [5]. Another GWAS study demonstrated that the SNP site rs346803513 in *DENND1A* is correlated with the disease pathogenesis of PCOS [28]. However, for Chinese women belonging to the Hui ethnic group, rs13405728 is found to be associated with PCOS. In contrast, no statistically significant correlation with PCOS is found for rs13429458 and rs2479106 in this ethnic group [29]. Two of the five SNPs (rs10986105 and rs10818854) on 9q33.3 are suggested to be associated with PCOS in the Chinese population [5]. Although the risk variant of rs2479106 has been successfully replicated in at least some of the Chinese populations, it is not found to be associated with PCOS in European cohorts [20]. Examination of the PCOS patients in this study indicates that the variant might be related to hyperandrogenism or irregular menses [20]. In European patients, one mistyped SNP (rs189947178, A/C) that may alter the structural conformation of the *DENND1A* protein is more prevalent in PCOS patients with moderate hirsutism [26]. Among the Caucasians, the rs2479106 G allele is associated with a decreased susceptibility to PCOS [21]. In the Bahraini Arabic population, the *DENND1A* SNPs including rs2479106, rs10986105 and rs10818854 are associated with PCOS [23]. It has been previously suggested that the association of rs10818854 and rs10986105 polymorphisms with PCOS is dependent on the ethnic origin of the population [12]. A meta-analysis showed that the DENND1A SNPs are associated with the pathogenesis of PCOS [30]. Again, a strong ethnic influence has been suggested, as rs2479106, rs10818854, and rs10986105 are associated with PCOS only in Asian populations, whereas rs10818854 and rs10986105 are correlated with PCOS in Caucasians. No correlation has been established between rs2479106 and PCOS.

This study focused on the five SNPs associated with the *DENND1A* gene among subjects in Eastern China in a large sample study; the results were persuasive and credible, thus filling the gap on the research of this area. The correlation between the *DENND1A* gene-related SNPs and PCOS incidence is subject to the ethnicity of the population and environmental modulation; whether our findings can be applied to populations other than Chinese or even Chinese in other geographic areas remains uncertain.

In this study, significant differences in LH levels and LH/FSH ratios of PCOS patients and controls were revealed based on the rs2479106 and rs2768819 polymorphisms (*P* < 0.05). *DENND1A*, which plays an important role in the expression and metabolism of hormones,, is associated with PCOS through the regulation of hyperandrogenemia [31]. On the other hand, the variation of *DENND1A* may affect the exocytosis of gonadotropins. *DENND1A* is also associated with androgen secretion and expression. The *DENND1A*.*V2* protein is increased in theca cells of PCOS patients [13]. Higher levels of *DENND1A*.*V2* in theca cells can promote *CYP17A1* and *CYP11A1* gene transcription and androgen synthesis, leading to hyperandrogenemia of PCOS [13]. In several studies, the association between PCOS and SNPs of *DENND1A* gene has been demonstrated and replicated. Taken together, these data strongly support a contributory role of *DENND1A* in the development of PCOS. Furthermore, it can be speculated that the *DENND1A* gene has an impact on the increase of androgen and the release of gonadotropin in PCOS patients.

## Conclusions

Our data provide further support for the established relationship between *DENND1A* polymorphisms and the pathogenesis of PCOS in a Chinese Han population from Eastern China. The tagging SNPs rs2479106 and rs2468819 in the *DENND1A* gene are associated with PCOS, whereas rs2670139, rs2536951 and rs2479102 are not correlated with PCOS in the same population. The sample size used in this study is not large enough, and may lead to limited power of the association test. We will continue collecting more samples and expect to have a larger sample size in future studies. Further research is required to fully reveal the mechanisms by which the tag SNPs in *DENND1A* may exert pathophysiological regulation in promoting or suppressing PCOS development. These observations and further clarifications may have both diagnostic and therapeutic implications for this disorder.

## Supplementary information

**Additional file 1: Figure S1.** A full picture of the LD pattern of *DENND1A* gene. The SNPs genotyped were tagging SNPs in *DENND1A* gene based on the Hap Map database (www.hapmap.org, Hap Map database release no. R2/phase III, population: CHB) for the Chinese Han population **Figure S1–1.** is the clear one, and the linkage association was marked in **Figure S1–2.** In Block 12, the pair-wise correlations between rs2479106 and the other four SNPs are the same (measured as D’ = 95%).

## Data Availability

The datasets generated and analysed during the current study are available in the NCBI BioSample & BioProject database repository. The data are accessible via the accession number: SAMN13640133 & PRJNA596822: human Phenotype or genotype (TaxID: 9606).

## Abbreviations

BMI: Body Mass Index
DENNDIA: DENN Domain Containing 1A
E2: Estradiol
ERAP1,: Endoplasmic Reticulum Amino Acid Peptidase 1
FSH: Follicle-Stimulating Hormone
LD,: Linkage Disequilibrium
LH: Luteinizing Hormone
LHCGR: Luteinizing Hormone/Choriogonadotropin Receptor
PCOS: Polycystic Ovary Syndrome
PRL: Prolactin
SNP: Single Nucleotide Polymorphism
T: Total Testosterone
THADA: Thyroid Associated Protein

## References

[1] Chen J, Shen S, Tan Y, Xia D, Xia Y, Cao Y, Wang W, Wu X, Wang H, Yi L (2015). The correlation of aromatase activity and obesity in women with or without polycystic ovary syndrome. J Ovarian Res.

[2] Azziz R, Woods KS, Reyna R, Key TJ, Knochenhauer ES, Yildiz BO (2004). The prevalence and features of the polycystic ovary syndrome in an unselected population. J Clin Endocrinol Metab.

[3] Costello MF, Eden JA (2003). A systematic review of the reproductive system effects of metformin in patients with polycystic ovary syndrome. Fertil Steril.

[4] Prapas N, Karkanaki A, Prapas I, Kalogiannidis I, Katsikis I, Panidis D (2009). Genetics of polycystic ovary syndrome. Hippokratia.

[5] Chen ZJ, Zhao H, He L, Shi Y, Qin Y, Shi Y, Li Z, You L, Zhao J, Liu J (2011). Genome-wide association study identifies susceptibility loci for polycystic ovary syndrome on chromosome 2p16.3, 2p21 and 9q33.3. Nat Genet.

[6] Shi Y, Zhao H, Shi Y, Cao Y, Yang D, Li Z, Zhang B, Liang X, Li T, Chen J (2012). Genome-wide association study identifies eight new risk loci for polycystic ovary syndrome. Nat Genet.

[7] Ke L, Che YN, Cao YX, Wu XK, Hu YL, Sun HX, Liang FJ, Sun J, Yi L, Wang Y (2010). Polymorphisms of the HSD17B6 and HSD17B5 genes in Chinese women with polycystic ovary syndrome. J Women's Health (Larchmt).

[8] Day FR, Hinds DA, Tung JY, Stolk L, Styrkarsdottir U, Saxena R, Bjonnes A, Broer L, Dunger DB, Halldorsson BV (2015). Causal mechanisms and balancing selection inferred from genetic associations with polycystic ovary syndrome. Nat Commun.

[9] Welt CK, Duran JM (2014). Genetics of polycystic ovary syndrome. Semin Reprod Med.

[10] Goodarzi MO, Jones MR, Li X, Chua AK, Garcia OA, Chen YD, Krauss RM, Rotter JI, Ankener W, Legro RS (2012). Replication of association of DENND1A and THADA variants with polycystic ovary syndrome in European cohorts. J Med Genet.

[11] Zhang YJ, Li L, Wang ZJ, Zhang XJ, Zhao H, Zhao Y, Wang XT, Li CZ, Wan JP. Association study between variants in LHCGR *DENND1A* and THADA with preeclampsia risk in Han Chinese populations. J Matern Fetal Neonatal Med. 2018:1–5. 10.1080/14767058.2018.1472228 29727258

[12] Dallel M, Sarray S, Douma Z, Hachani F, Al-Ansari AK, Letaifa DB, Mahjoub T, Almawi WY (2018). Differential association of *DENND1A* genetic variants with polycystic ovary syndrome in Tunisian but not Bahraini Arab women. Gene.

[13] McAllister JM, Modi B, Miller BA, Biegler J, Bruggeman R, Legro RS, Strauss JF (2014). Overexpression of a *DENND1A* isoform produces a polycystic ovary syndrome theca phenotype. Proc Natl Acad Sci U S A.

[14] Cui L, Zhao H, Zhang B, Qu Z, Liu J, Liang X, Zhao X, Zhao J, Sun Y, Wang P (2013). Genotype-phenotype correlations of PCOS susceptibility SNPs identified by GWAS in a large cohort of Han Chinese women. Hum Reprod.

[15] Casarini L, Simoni M, Brigante G (2016). Is polycystic ovary syndrome a sexual conflict? A review. Reprod Biomed Online.

[16] Marat AL, Dokainish H, McPherson PS (2011). DENN domain proteins: regulators of Rab GTPases. J Biol Chem.

[17] Allaire PD, Ritter B, Thomas S, Burman JL, Denisov AY, Legendre-Guillemin V, Harper SQ, Davidson BL, Gehring K, McPherson PS (2006). Connecdenn, a novel DENN domain-containing protein of neuronal clathrin-coated vesicles functioning in synaptic vesicle endocytosis. J Neurosci.

[18] Allaire PD, Marat AL, Dall'Armi C, Di Paolo G, McPherson PS, Ritter B (2010). The Connecdenn DENN domain: a GEF for Rab35 mediating cargo-specific exit from early endosomes. Mol Cell.

[19] Olszanecka-Glinianowicz M, Banas M, Zahorska-Markiewicz B, Janowska J, Kocelak P, Madej P, Klimek K (2007). Is the polycystic ovary syndrome associated with chronic inflammation per se?. Eur J Obstet Gynecol Reprod Biol.

[20] Welt CK, Styrkarsdottir U, Ehrmann DA, Thorleifsson G, Arason G, Gudmundsson JA, Ober C, Rosenfield RL, Saxena R, Thorsteinsdottir U (2012). Variants in *DENND1A* are associated with polycystic ovary syndrome in women of European ancestry. J Clin Endocrinol Metab.

[21] Eriksen MB, Brusgaard K, Andersen M, Tan Q, Altinok ML, Gaster M, Glintborg D (2012). Association of polycystic ovary syndrome susceptibility single nucleotide polymorphism rs2479106 and PCOS in Caucasian patients with PCOS or hirsutism as referral diagnosis. Eur J Obstet Gynecol Reprod Biol.

[22] Gao J, Xue JD, Li ZC, Zhou L, Chen C (2016). The association of *DENND1A* gene polymorphisms and polycystic ovary syndrome risk: a systematic review and meta-analysis. Arch Gynecol Obstet.

[23] Gammoh E, Arekat MR, Saldhana FL, Madan S, Ebrahim BH, Almawi WY (2015). *DENND1A* gene variants in Bahraini Arab women with polycystic ovary syndrome. Gene.

[24] Bao S, Cai JH, Yang SY, Ren Y, Feng T, Jin T, Li ZR (2016). Association of *DENND1A* gene polymorphisms with polycystic ovary syndrome: a meta-analysis. J Clin Res Pediatr Endocrinol.

[25] Lerchbaum E, Trummer O, Giuliani A, Gruber HJ, Pieber TR, Obermayer-Pietsch B (2011). Susceptibility loci for polycystic ovary syndrome on chromosome 2p16.3, 2p21, and 9q33.3 in a cohort of Caucasian women. Horm Metab Res.

[26] Eriksen MB, Nielsen MF, Brusgaard K, Tan Q, Andersen MS, Glintborg D, Gaster M (2013). Genetic alterations within the *DENND1A* gene in patients with polycystic ovary syndrome (PCOS). PLoS One.

[27] Malini NA, Roy GK (2018). Evaluation of different ranges of LH:FSH ratios in polycystic ovarian syndrome (PCOS) - clinical based case control study. Gen Comp Endocrinol.

[28] Chen L, Hu LM, Wang YF, Yang HY, Huang XY, Zhou W, Sun HX (2017). Genome-wide association study for SNPs associated with PCOS in human patients. Exp Ther Med.

[29] Ha L, Shi Y, Zhao J, Li T, Chen ZJ (2015). Association study between polycystic ovarian syndrome and the susceptibility genes polymorphisms in Hui Chinese women. PLoS One.

[30] Danhong P, Luo J, Li L, Xuelin L, Mulan R (2017). Meta-analysis on relationship between *DENND1A* polymorphisms and polycystic ovary syndrome. J Nanjing Med Univ.

[31] Saxena R, Georgopoulos NA, Braaten TJ, Bjonnes AC, Koika V, Panidis D, Welt CK (2015). Han Chinese polycystic ovary syndrome risk variants in women of European ancestry: relationship to FSH levels and glucose tolerance. Hum Reprod.

