# Supplementary figures and images for: Association analysis between the tag single nucleotide polymorphisms of DENND1A and the risk of polycystic ovary syndrome in Chinese Han women
Source: BMC Med Genet. 2020 Jan 15;21:14. doi: 10.1186/s12881-019-0945-1 (PMC6964046; doi:10.1186/s12881-019-0945-1)

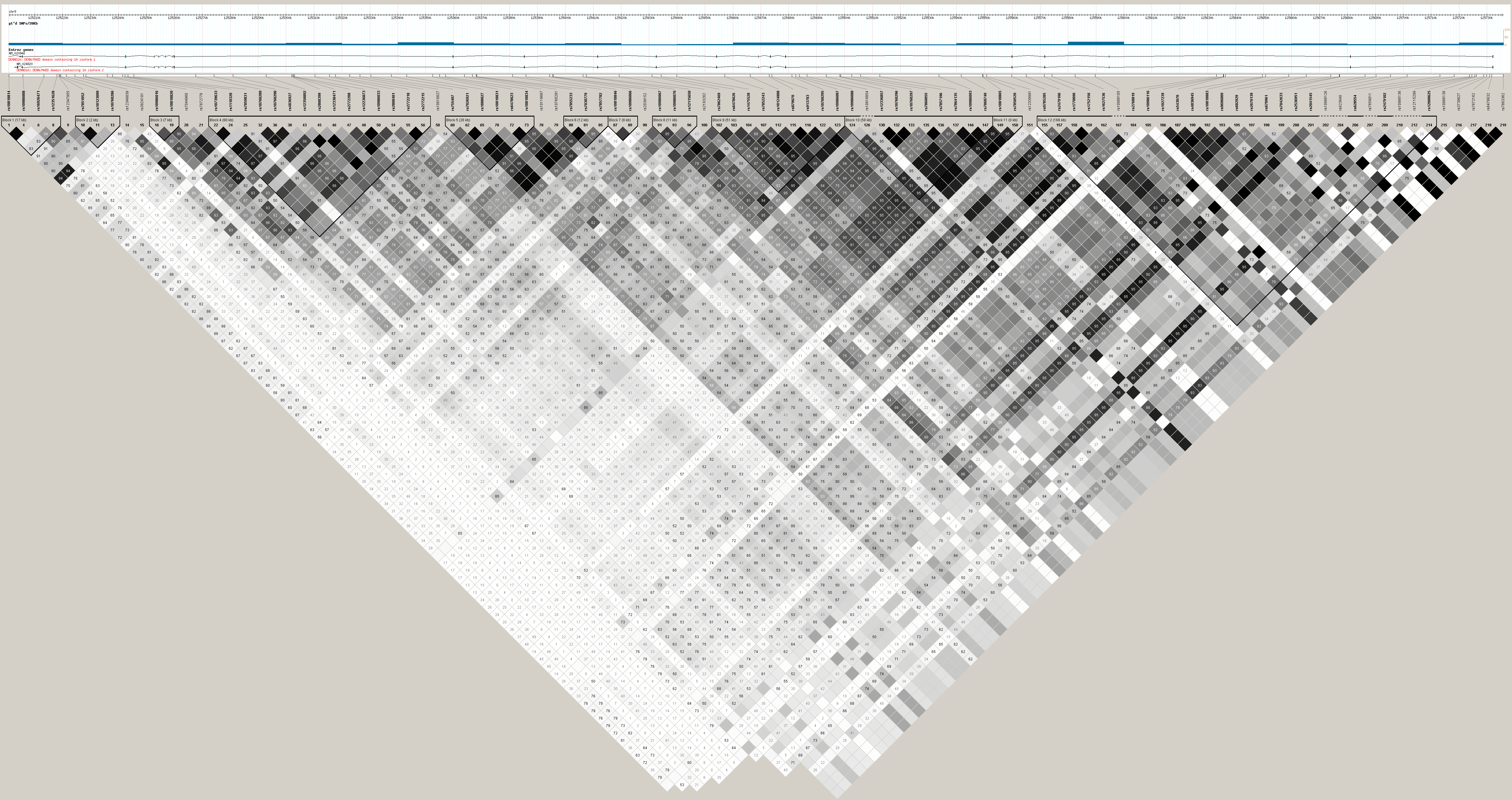

Supplement: Supplementary file 1 — Additional file 1: Figure S1. A full picture of the LD pattern of DENND1A gene. The SNPs genotyped were tagging SNPs in DENND1A gene based on the Hap Map database (www.hapmap.org, Hap Map database release no. R2/phase III, population: CHB) for the Chinese Han population Figure S1–1. is the clear one, and the linkage association was marked in Figure S1–2. In Block 12, the pair-wise correlations between rs2479106 and the other four SNPs are the same (measured as D’ = 95%). [file 12881_2019_945_MOESM1_ESM.zip › Supplemental Fig. 1-1R6.png]

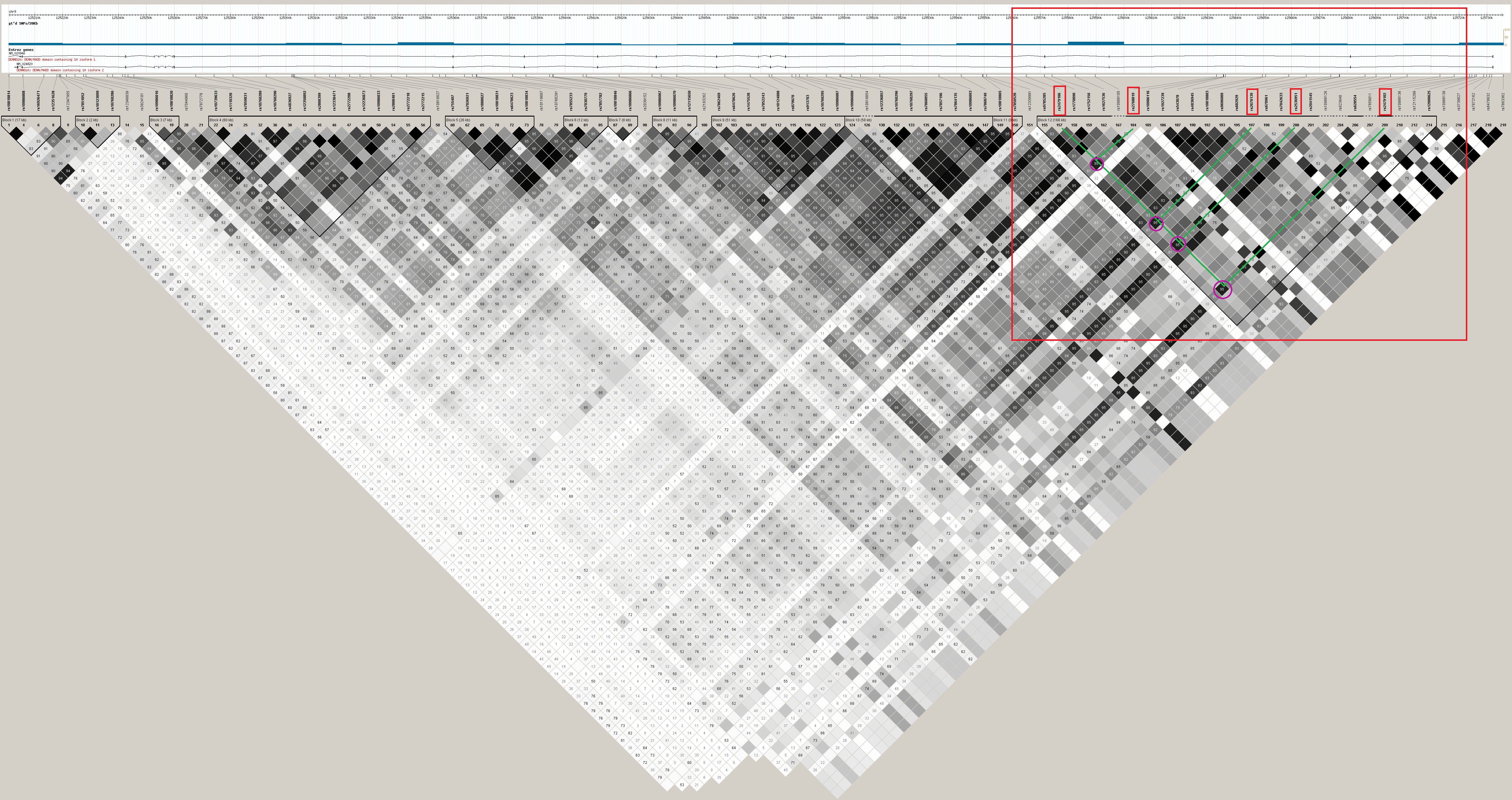

Supplement: Supplementary file 1 — Additional file 1: Figure S1. A full picture of the LD pattern of DENND1A gene. The SNPs genotyped were tagging SNPs in DENND1A gene based on the Hap Map database (www.hapmap.org, Hap Map database release no. R2/phase III, population: CHB) for the Chinese Han population Figure S1–1. is the clear one, and the linkage association was marked in Figure S1–2. In Block 12, the pair-wise correlations between rs2479106 and the other four SNPs are the same (measured as D’ = 95%). [file 12881_2019_945_MOESM1_ESM.zip › Supplemental Fig. 1-2 with markerR6.png]
